# An fMRI study of nonverbally gifted reading disabled adults: has deficit compensation effected gifted potential?

**DOI:** 10.3389/fnhum.2013.00507

**Published:** 2013-08-29

**Authors:** Jeffrey W. Gilger, Thomas M. Talavage, Olumide A. Olulade

**Affiliations:** ^1^Psychological Sciences, University of California MercedMerced, CA, USA; ^2^Weldon School of Biomedical Engineering, Purdue UniversityWest Lafayette, IN, USA; ^3^The School of Electrical and Computer Engineering, Purdue UniversityWest Lafayette, IN, USA; ^4^Center for the Study of Learning, Department of Pediatrics, Georgetown University Medical CenterWashington, DC, USA

**Keywords:** giftedness, reading disability, twice-exceptional, neuroimaging, spatial visualization

## Abstract

Neuroscience has advanced our understanding of the neurological basis of reading disability (RD). Yet, no functional imaging work has been reported on the twice-exceptional dyslexic: individuals exhibiting both non-verbal-giftedness and RD. We compared groups of reading-disabled (RD), non-verbally-gifted (G), non-verbally-gifted-RD (GRD), and control (C) adults on validated word-rhyming and spatial visualization fMRI tasks, and standardized psychometric tests, to ascertain if the neurological functioning of GRD subjects was similar to that of typical RD or G subjects, or perhaps some unique RD subtype. Results demonstrate that GRD adults resemble non-gifted RD adults in performance on paper-and-pencil reading, math and spatial tests, and in patterns of functional activation during rhyming and spatial processing. Data are consistent with what may be a shared etiology of RD and giftedness in GRD individuals that yields a lifespan interaction with reading compensation effects, modifying how their adult brain processes text and spatial stimuli.

## Introduction

Reading disability (RD) is perhaps the most heavily studied of the developmental learning disorders, affecting approximately 7–10% of the school-aged population. RD is also the most common learning disability, with ~85% of the learning disabled (LD) population having a reading-related condition (Lerner, 1989). While a lot is known about RD alone, the systematic and empirical study of giftedness alongside RD, and twice-exceptionality (i.e., a specific learning disability concomitant with a cognitive gift or talent), is relatively lacking.

Prevalence rates of twice exceptionality in school-age children vary widely depending on sample characteristics, the disabilities being considered, and definitions of categories. Some of the best estimates have placed these rates for heterogeneous LD child populations at around 2–5% (Ruban and Reis, 2005). This represents a relatively high rate in practice or in the classroom. Given that reading-related problems are quite common in LD populations (Lerner, 1989), many gifted-LD children might be classified as having reading disabilities as part of their profile. At the moment, however, the actual rates of gifted-RD (GRD) remain a mystery.

Neuroscience research on the GRD student is required so that we may better understand this condition, and improve current approaches to diagnosis and treatment. Practitioners and clinicians agree that the GRD individual can manifest different behavioral symptoms than the individual with RD or giftedness alone (Eide and Eide, 2006; Foley Nicpon et al., 2011; McClain and Pfeiffer, 2012): GRD individuals are often lost in the school or IEP system, often have their talents neglected in favor of remediation, and confuse diagnosticians such that they do not qualify for reading services or gifted programs, among other complications. Thus, in summary, twice exceptional individuals pose complicated diagnostic and educational problems, as well as a neurological paradox in need of exploration.

There exists a long history of interest in the gifted RD individual. Some have extolled the virtues of being RD in general, and claim that there are shared etiologies linking RD and, particularly, non-verbal giftedness. Accordingly, famous and successful RD business leaders, artists, and scientists are often cited, and the RD individual is viewed as an “untapped resource” with special skills useful to society. Although some research has demonstrated that RD is overrepresented in more spatially oriented or non-verbally gifted populations and associated careers (for example, Art, Mathematics, Architecture, and Physics among others; West, 1999; Winner et al., 2001; Eide and Eide, 2006; Schneps et al., 2007; Logan, 2009), related experimental studies that have asked if non-verbal skills, such as spatial visualization, are elevated in RD samples, have yielded mixed and largely negative results (Winner et al., 2001). Still, there exists some theory that would support a “*shared etiology hypothesis*,” and predicts that RD and non-verbal giftedness should co-occur more often than chance expectations (Geschwind and Behan, 1982; Geschwind and Galaburda, 1987; Galaburda, 1992; Newman and Sternberg, 2004; Craggs et al., 2006; Gilger and Hynd, 2008). Geschwind and colleagues, for example, discussed the potential etiologic relationships between giftedness and RD over 30 years ago, and gave a tentative theory to account for the (possible) overrepresentation of non-verbal gifts in RD samples (as well as other conditions such as left handedness and immune system dysfunction). Aspects of these theories in humans, however, have not been well-assessed, or when tested, have yielded inconsistent results (e.g., McManus and Bryden, 1991; Bryden et al., 1994).

If RD individuals are indeed over represented in non-verbal careers, or more often excel in the processing of non-verbal material, they might do so for two reasons (Winner et al., 2001). One possibility discussed above is that there is a shared etiology between RD and giftedness such that the two conditions co-occur more often than would be expected by chance. Another reason is that the RD individual compensates for verbal weaknesses by selecting and practicing skills in non-verbal domains, thus increasing their presence in such fields, and improving related cognitive abilities.

### Current study

There are currently no published neuroscientific studies of the twice-exceptional, or gifted RD samples. This first study focuses on the ways in which neural function in GRD subjects differs (or not) from that of subjects that have a diagnosis of giftedness or RD alone, using tasks that assess the most commonly discussed twice-exceptional dyad of ability vs. disability in persons with a reading problem: high spatial/non-verbal skills vs. low reading/verbal skills. Indeed, prior research exists that has examined this dyad of skills separately. First, there is research documenting how the brains of typical readers and the RD function during word reading and language-oriented tasks (Pugh et al., 2001; Shaywitz et al., 2002): a basic model primarily includes left hemisphere inferior frontal gyrus (IFG) (articulatory mapping), the superior temporal gyrus (STG) (letter-sound correspondences), the angular gyrus (modality coordination), and the occipitotemporal region (graphemic analysis). Commonly observed in RD individuals is reduced activity in the superior temporal and occipitotemporal areas, and an overactivation in the inferior frontal areas, although this may vary with age and degree of remediation (Simos et al., 2002).

Other research has examined the spatial rotation/visualization portion of the dyad in gifted and non-gifted subjects (O'Boyle et al., 2005; Gogos et al., 2010), reporting in general, greater bilateral activation in gifted subjects relative to controls, particularly in the posterior parietal and occipital lobes, along with a decrease in activation in “decision-making” areas of the frontal lobes. When spatial problems are simple or do not require dynamic mental manipulation, gifted subjects show less activation than controls, perhaps indicating more efficient processing.

In light of our understanding how RD and spatially-gifted brains function when processing spatial or reading stimuli separately, we examined the neural function of GRD individuals in response to spatial rotation and reading tasks, and assessed if activation patterns matched one of several hypotheses: A first hypothesis was that *brain activity in GRD individuals represents an independent admixture of gifted (G) and RD activation patterns*. This predicted the typical activation patterns seen in RD individuals during word reading (e.g., decreased temporo-parietal and temporo-occipital activity), and increased bilateral activation during spatial rotation (e.g., in the parietal-occipital areas) as observed in gifted individuals, and would suggest that the neural mechanisms underlying giftedness and RD in these individuals interact only minimally. An alternative hypothesis was that *GRD brains activate like RD brains*. If this were the case, then we predicted decreased activation in word reading areas, as well as activation similar to, or less than, normal controls during spatial processing. A variant of this second hypothesis would be the opposite case, where GRD individuals exhibit brain activation patterns similar to those of gifted-only individuals, and underactivation is not observed in the aforementioned language related regions during reading tasks. Finally, a third hypothesis was that *GRD brains are unique brains and activate in ways that are deviant from G or RDs alone or in combination*, suggesting that the neural mechanisms underlying giftedness and RD interact in a manner to produce a unique pattern of activation.

## Materials and methods

### Participants

Twenty-nine English speaking subjects (14 female) with no previous diagnosis of neurological or psychiatric disorder were included in final analysis, after eight of the original sample were excluded due to excessive head motion during the MRI scan, incomplete data, or if they did not meet psychometric criteria to be included in one of the four matched groups. Subjects were placed into one of four groups based on the following criteria: *Reading disabled or RD* (*N* = 6/3F): Full-Scale IQ (FSIQ) standard score above 85 with Verbal IQ (VIQ) and Performance IQ (PIQ) in the normal range (mean = 100, with a standard deviation of 15), and a previous diagnosis of RD and/or significant underachievement in reading and/or spelling that began during the elementary or middle school grades. Subjects also had confirmed diagnoses of a disability through the university testing requirement to be qualified for learning disability services. Psychometric tests conducted as part of this study had to confirm an RD profile; *Non-verbally gifted or G* (*N* = 5/1F): FSIQ and VIQ in the normal range and a PIQ in the Superior or better range (above 120)^1^. Subjects in this group had no history of learning problems and demonstrated at least normal abilities in our psychometric testing; *Non-verbally gifted and reading disabled or GRD* (*N* = 9/4F)^1^: This group met the IQ criteria for the G group and also had a history or university documentation indicating a reading disorder that was supported by our psychometric testing. *Controls or C* (*N* = 9/6F): These subjects were normal readers with IQs in the normal range and no history of learning difficulties according to self-report and supported by our psychometric testing. The disparity in male/female ratios for the G group relative to all others was noted, thus gender was added as a regressor of no-interest in fMRI analysis to remove any effect of this potential confound.

At the time of the study, subjects were college students at a large Midwestern Research institution. Experimental procedures were approved by the University Institutional Review Board, written informed consent was obtained from each subject, and subjects were compensated for their participation. It is important to point out that the C, G, RD, and GRD samples were carefully selected such that they were matched as much as possible on verbal and full scale IQs, and that the RD and GRD groups presented a reading test profile consistent with a diagnosis of a reading-specific disorder. The recruitment procedure involved interviewing each potential subject in-person. If the interview indicated that the person might fit the criteria of one of the four groups, he or she was then given an IQ test. If the results of the IQ test were within required ranges, the person was advanced to the psychometric portion of the study that included the reading tests and other measures. Finally, those that met the requirements to be classified as C, RD, G, or GRD were admitted into the MRI portion of the study. Overall, ~35% of the subjects interviewed were ultimately given an MRI. While our ultimate sample sizes for each group may be small, this is to be expected given the rarity of the samples, and what was required for the matching criteria. We acknowledge that observed results may be specific to our sample and will require larger sizes for population inference.

### Behavioral battery

Participants underwent a battery of behavioral/psychometric tests that were used to confirm diagnoses and group placement (see Table 1): the Wechsler Abbreviated Scale of Intelligence (WASI; Wechsler, 1999), comprised of the Vocabulary, Similarities, Block Design and Matrices subtests. The WASI yields highly reliable Verbal (VIQ), Performance (PIQ) and Full Scale IQs (FSIQ); the Reading Fluency (WJRF: reading of sentences), Passage Comprehension (WJPC: contextual understanding of a written passage), and Spatial Relations (WJSR: detection of object features, mental manipulation of objects and visual matching) subtests of the Woodcock-Johnson Test of Achievement (Woodcock et al., 2001); the Rapid Automatized Naming tests that assess fluency for reading letters and numbers (RAN; Wolf and Denckla, 2005); and the Word Recognition (WRATREC: single word reading), Spelling (WRATSPELL: written spelling of orally presented words), Mathematics Computation (WRATMATH: written math calculations), and Sentence Completion (WRATSC: written sentence comprehension) subtests of the Wide Range Achievement Test (Wilkinson and Robertson, 2006). Other self-report questionnaire and interview data, not relevant to this report, were collected as well. The RD and GRD samples were required to have been qualified for services for RD at the university. A licensed psychologist performed testing at the university within 2 years of the start of this study, and resulting data was made available to our research team.

**Table 1:** **Descriptive data for controls (C), Reading disabled (RD), Gifted (G), and Gifted-reading disabled (GRD) subjects^a^**.

	**Mean (*SD*)**				***t*-Value (relative to C group)**			***t*-Value 2-tailed significance^b^**		
	***C***	**RD**	**GRD**	***G***	**RD**	**GRD**	***G***	**RD**	**GRD**	***G***
AGE	20.6 (1.33)	20.7 (1.21)	22.1 (4.09)	20.4 (1.52)	−0.16	−1.12	0.06	0.87	0.30	0.96
WASI VIQ^c^	103.6 (6.98)	99 (11.61)	103.6 (14.65)	103.8 (4.15)	0.96	−0.01	−0.07	0.36	0.99	0.95
WASI PIQ	108.7 (5.97)	109.5 (11.19)	125.8 (3.77)	126.6 (4.04)	−0.19	−6.94	−5.94	0.85	0.00	0.00
WASI FSIQ	106.0 (4.86)	104.5 (11.54)	115.4 (10.99)	115.6 (2.61)	0.56	−2.50	−3.68	0.59	0.04	0.00
WRAT WRec^c^	111.7 (10.24)	91.3 (11.38)	97.1 (14.42)	120.8 (17.79)	3.61	2.42	−1.24	0.00	0.03	0.24
WRAT SC	109.0 (10.55)	99.5 (13.49)	107.7 (11.95)	116.2 (10.18)	1.53	0.20	−1.24	0.15	0.84	0.24
WRAT Spell	112.9 (11.74)	89.5 (12.92)	92.9 (7.78)	118.0 (20.21)	3.58	4.26	−0.52	0.00	0.00	0.64
WRAT Math	114.9 (13.04)	109.5 (16.89)	113.1 (15.19)	123.6 (9.74)	0.70	0.26	−1.29	0.50	0.80	0.22
RAN L^d^	111.8 (4.76)	101.2 (5.42)	97.8 (9.67)	108.8 (6.61)	4.00	3.49	0.98	0.00	0.00	0.38
RAN 2LN	117.2 (8.47)	102.0 (10.43)	97.1 (17.72)	111.6 (4.56)	3.12	2.76	1.36	0.00	0.02	0.19
RAN 3LN	117.7 (8.60)	95.3 (14.24)	93.4 (23.72)	114.8 (8.87)	3.81	2.61	0.59	0.00	0.03	0.57
WJ RF^c^	119.0 (9.77)	96.7 (5.99)	89.5 (9.56)	104.4 (6.87)	4.97	1.94	2.94	0.00	0.00	0.01
WJ PC	103.9 (5.40)	98.3 (6.53)	104.4 (7.30)	113.8 (7.43)	1.80	−0.17	−2.89	0.10	0.87	0.01
WJ SR	105.6 (7.40)	103.7 (6.74)	109.4 (7.85)	117.8 (8.23)	0.50	−1.02	−2.76	0.63	0.34	0.03
MRI Rhyming (%Acc.)^e^	89.9 (3.33)	77.4 (7.11)	76.9 (9.94)	88.6 (4.64)	4.01	3.52	0.57	0.00	0.00	0.58
MRI Rhyming (RT)	1553.1 (339.4)	2052.7 (478.9)	1976.5 (184.9)	1692.6 (275.0)	−2.21	−3.17	−0.83	0.06	0.00	0.42
MRI Spatial (% Acc.)	81.5 (8.4)	71.1 (18.7)	83.2 (4.1)	90.4 (3.7)	1.28	−0.53	−2.73	0.24	0.62	0.02
MRI Spatial (RT)^f^	2192.4 (314.7)	2338.3 (528.1)	2238.3 (190.6)	2076.4 (291.4)	−0.61	−0.37	0.69	0.56	0.73	0.51

a *Number of subjects in all analyses: 9 C, 6 RD, 8 GRD, 5 G*.

b *Where variances were unequal, corrections were applied to t/d.f.'s. All t-tests on 13 d.f*.

c *WASI IQs, WRAT subtests and WJ subtests are age corrected standard scores with mean of 100, standard deviation of 15*.

d *RAN composites created as per manual in age corrected standard score form with mean of 100 and standard deviation of 15; RAN L: Rapid Automatized Naming for Letters; RAN 2 LN and RANN 3LN are composites that include RAN letters and numbers stimuli (see Wolf and Denckla, 2005)*.

e *MRI task performance data are in percent correct*.

f *MRI task reaction time data are in ms*.

### fMRI acquisition and task

During a single continuous MRI session lasting ~90-min, subjects completed two (separate) block-design runs each of a written language/reading assessment, and a spatial visualization assessment. The reading assessment consisted of a rhyme judgment task. In each trial, subjects were asked to indicate whether a pair of displayed words (W; e.g., sleet and cleat) pseudo or nonwords (NW; e.g., mucit and rucket) rhymed (Pugh et al., 2000). Each block consisted of ten pseudo-randomized trials during which the stimulus was displayed for a maximum of 3.5 s, followed by a fixation crosshair for 0.5 s. If the subject responded prior to the allotted time, the stimulus was removed and replaced with the fixation crosshair for the remainder of the trial. This was done to ensure that resulting neural activations would be specific to the active performance of the task (e.g., rhyming), and not simply passive observation of the stimuli, and we accounted for partial volumes as a result of the variable stimulus presentation length in all subsequent fMRI analyses. Each run consisted of four W-blocks and four NW-blocks.

The spatial assessment was based on the mental rotation task of Shepard and Metzler (Shepard and Metzler, 1971). Here, subjects were required to determine whether two objects displayed on the screen were in the same orientation, or were mirror opposites and thus, different. These stimuli were again presented using a block design paradigm. In half of the blocks, the object on the left was displayed so that it's vertical axis was straight while the second object was rotated along either the X, Y, or Z axis at one of nine possible angles between 20 and 180° (in 20° increments), and subjects were required to rotate one object with respect to the other in order to make the determination (R). In the remaining blocks, the two objects were displayed at the same angle and no rotation was required (NR). Each run consisted of three R-blocks and three NR-blocks. Each block consisted of 8 trials during which the stimulus was displayed for a maximum of 4.5 s followed by a fixation crosshair for 0.5 s.

**Figure 1:**
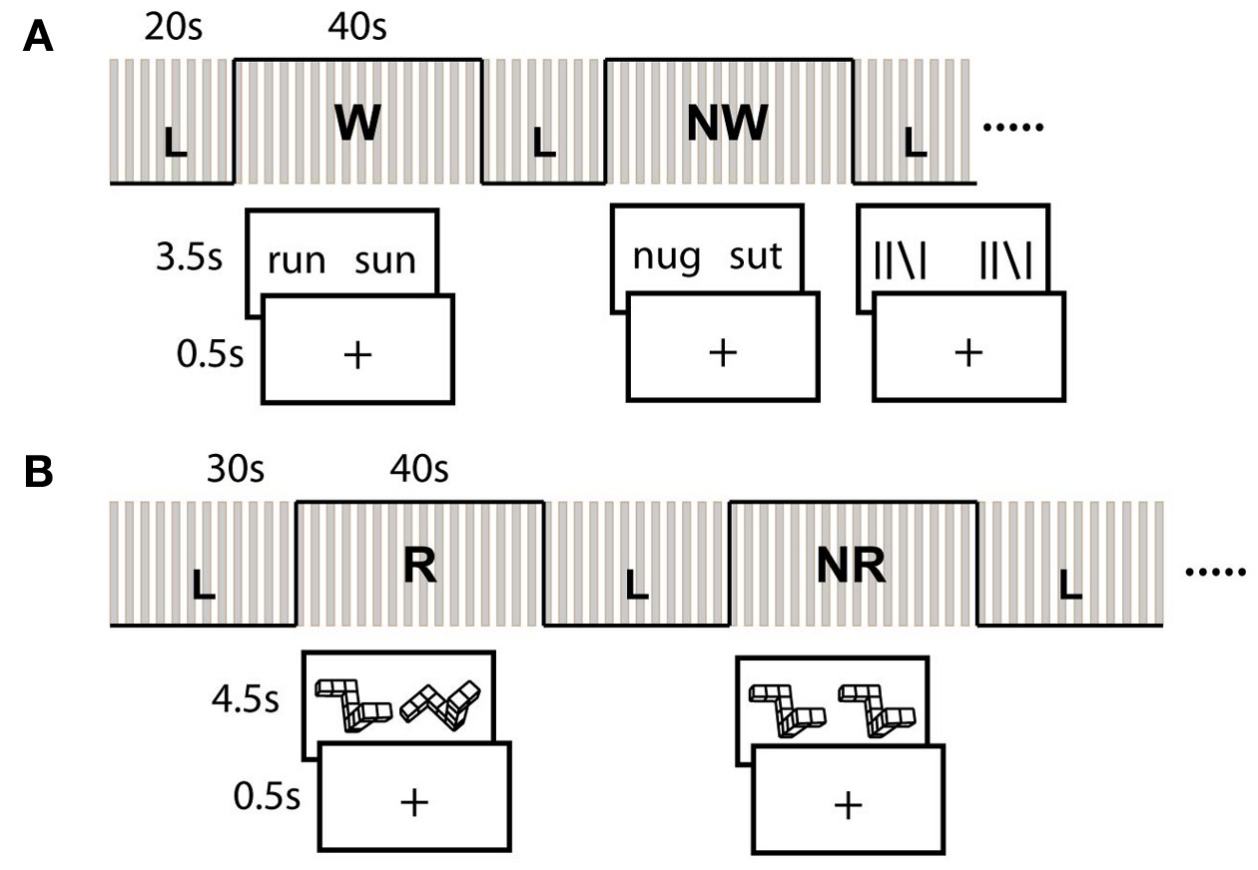
**fMRI Tasks and block design paradigm. (A)** Verbal Tasks involved rhyme judgment for real word (W) and pseudo-word (NW) pairs. **(B)** Spatial tasks were adapted from Shepard and Metzler cubes and involved same/different judgment. Certain blocks required mental rotation (R) of the object to make this determination. Line control (L) task also involved same/different judgment. Gray bars represent timing of image acquisition for each block.

The same standard line judgment (L) control condition (Shaywitz et al., 1998; Pugh et al., 2000) was utilized for both the reading and spatial assessments. This condition was presented in blocks that were interspersed between the task blocks (i.e., W/NW and R/NR), and served as a baseline subtraction in each case to control for basic visual attention, motor (button responses), and decisional activation patterns not of interest to this work. During this condition, subjects were asked to indicate whether two sets of lines displayed on the screen were exactly identical (i.e., in terms of orientation at all positions). The length of the control block was 20 s for the word reading assessment, and 30 s for the spatial assessment. See Figure 1 for a diagram of the blocking and example stimuli.

Throughout the experiments, all stimuli were displayed in black and rear-projected onto a blank white background using an NEC MultiSync LT81 projector. Subjects were asked to respond as quickly as possible to each trial with the index (“yes”) and middle (“no”) fingers of their self-reported dominant hand using a fiber optic response pad. There were 80 total trials each of the W and NW task, and 48 trials each of the R and NR task. We acquired data using a 3T GE Signa HDx scanner (Purdue University MRI Facility, West Lafayette, IN). Functional images consisted of 44 contiguous axial slices covering the whole brain acquired with the following parameters: field of view = 240 mm, slice thickness = 2.5 mm (0.5 mm inter-slice gap), in-plane resolution = 64 × 64, flip angle = 80°, *TE* = 22 *ms*, *TR* = 2.5 s.

### Analysis

Pre-processing and analysis of functional datasets was performed using SPM8 (http://fil.ion.ucl.ac.uk/spm/). To prevent T1 saturation effects, the first 4 scans of each run were discarded prior to pre-processing. The resulting datasets were subsequently motion corrected, normalized to the Montreal Neurological Institute (MNI) EPI template, re-sampled to 2 mm^3^ isotropic voxels, and smoothed with a Gaussian kernel of 8 mm full width at half maximum. Datasets were subsequently examined for head motion artifacts. Time-points for which the scan-to-scan motion was greater than a pre-determined threshold of 0.75 mm were removed from further analysis. Subjects for whom more than 20% of the images in the run exhibited scan-to-scan motion beyond the specified threshold were excluded from further analysis.

Statistical analysis at the first level involved generating parametric activation maps for the individual subjects in each group using the canonical SPM hemodynamic response function. Functional datasets at the first level were high-pass filtered with a cut-off of 128 s, and corrected for auto-correlations using an AR(1) model (Friston et al., 2002). For the statistical analysis in this study, we combined W and NW trials for the reading assessment and R and NR trials for the spatial assessment, as we were interested in the overall differences during word (whether or not subjects were faced with words or non-words) and spatial processing (whether or not subjects were faced with rotate or non-rotate stimuli). In addition, the stimulus types within the word-reading or spatial tasks have previously been shown to elicit activity in similar regions (Olulade et al., 2012), and combination of the W and NW and the R and NR trials in this manner served to improve our statistical power and simplify analyses^2^. Thus, conditions of interest were defined as ***Rhyming*** vs. control [i.e., (W + NW) vs. L] for the reading assessment, and ***Spatial*** vs. control [i.e., (R + NR) vs. L] for the spatial assessment.

Given that the stimulus was removed and replaced with a fixation crosshair following the subjects' response, the design matrix was adapted such that only volumes during which the stimulus was still being displayed were included in the analysis. None of these volumes were excluded due to motion in any subjects, as scan-to-scan motion for these did not exceed the aforementioned pre-specified threshold. Group random effects activation maps for each of the aforementioned contrasts were generated using the subject-specific contrast images in an ANOVA: Full Factorial design: A × B; *A = Group* (Between-subject factor: RD, C, GRD, G); *B = Subject* ([1 … n]—random). In parallel with the single-subject analysis, group level contrasts were defined as ***Rhyming*** vs. ***L*** and ***Spatial*** vs. ***L*** for each of the four groups. This ANOVA was conducted separately for the reading and spatial assessments. Subject gender and average reaction time were included as co-variates of no-interest for this portion of the analysis. Significantly active clusters were considered to be those that survived a cluster-size whole brain correction of *p* < 0.05, at a cluster-defining threshold of *p* < 0.001, implemented using the CorrClusTh algorithm by Nichols: (http://www.sph.umich.edu/~nichols/JG2/CorrClusTh.m).

To examine brain regions that exhibited reliable differences between the four groups, we tested for a significant main effect of *Group* (cluster-defining threshold = *p* < 0.001; cluster size corrected threshold = *p* < 0.05) from the aforementioned ANOVA. This analysis was again performed separately for the rhyming and spatial assessments. In each case, significantly active clusters were extracted as regions of interest (MarsBar: http://marsbar.sourceforge.net). Percent signal change was calculated for each subject within these regions for both the reading and spatial assessments, and averaged to obtain a group mean. *Post-hoc* comparisons were made between the GRD group and either the RD group or the G group, and in some cases with the control group.

## Results

### Behavioral battery

The descriptive data are presented in Table 1. Statistical tests were performed comparing controls (C) to each of the three other groups. The grouping manipulation was successful: mean VIQ and FSIQs were in the normal range for all four groups, while the G and GRD groups excelled on mean PIQ. The significant though small elevation in FSIQ for the G and GRD groups is due to their high PIQ and is unavoidable given how FSIQ is calculated. However, the groups were well-matched on VIQ. The RD and GRD groups performed significantly poorer on the reading-spelling accuracy and fluency tests (WRATREC, WRATSPELL, RANL, RAN2LN, RAN3LN, WJRF) that we administered. Reading comprehension was not significantly different, likely because these were college students, and the pattern of word recognition and fluency for the RD and GRD groups is consistent with expectations for educationally advanced and “compensated” RD adults (Wolfe et al., 2008). The G group performed at a slightly lower level on fluency than the C group (WJRF), and better than the C group on measures of comprehension (WJPC) and spatial relations (WJSR).

Our key sample selection variables were presence or absence of RD, and performance on the PIQ test. Based on prior literature we expected RD to be associated with fMRI word rhyming task performance (e.g., Pugh et al., 2000) and PIQ to be associated with fMRI spatial rotations task performance (e.g., O'Boyle et al., 2005). As predicted and displayed in Table 2, our three main reading variables, WRATREC, WRATSPELL, and WJRF, were not correlated with fMRI spatial task accuracy performance (*p* > 0.10), but were correlated with accuracy on the real word and pseudo-word fMRI rhyming task. Also, according to expectation, PIQ was not correlated with performance accuracy for the fMRI rhyming tasks, but was significantly correlated with accuracy on the fMRI spatial tasks (as well as with the WJSR). It is noted that here we present data separately for within-task stimulus types (W and NW and R and NR), whereas these stimulus types were combined for other analyses. The similar pattern of correlations across with-task stimulus type, suggests that combining stimulus types as we have done for all other analyses was not unreasonable.

**Table 2:** **Correlations among PIQ, reading and fMRI task accuracy^1^**.

	**Rhyming task**		**Spatial task**	
	**Word**	**Non-word**	**Rotate**	**Non-rotate**
PIQ	−0.04	−0.23	0.48^**^	0.45^**^
WRATWR	0.48^**^	0.51^**^	0.26	0.30
WRATS	0.59^**^	0.61^**^	0.14	0.34
WJRF	0.43^*^	0.56^**^	−0.02	0.27

** p < 0.01, two-tailed;

* *p < 0.05, two-tailed*.

1 *Number of subjects in all analyses: 28–29, due to occasional missing data. PIQ, WRAT, and WJ subtests were in age-corrected standard score form when correlated with fMRI accuracy rates*.

ANOVA on the in-scanner accuracy revealed a significant main effect of *Group* for both the rhyming [*F*_(3, 25)_ = 12.4; *p* < 0.001] and spatial tasks [*F*_(3, 25)_ = 5.01; *p* = 0.004]. Again, in accordance with predictions, significantly poorer accuracy performance was exhibited by the RD and GRD subjects relative to controls for the fMRI rhyming task (Pugh et al., 2000). In addition, accuracy performance for the fMRI spatial task was superior for the G individuals. It is noteworthy (see discussion below) that the GRD group did not show superior performance akin to the GRD group on the fMRI spatial task even though PIQ was correlated with fMRI spatial task accuracy.

ANOVA on the in-scanner reaction times revealed a significant main effect of *Group* for the rhyming task [*F*_(3, 25)_ = 7.28; *p* < 0.001]. GRD and RD subjects responded slower to these stimuli than the other groups. No effect was observed for RT on the spatial task [*F*_(3, 25)_ = 0.84; *p* = 0.480]. Overall, our mean psychometric, correlational, and fMRI accuracy data suggest successful group formation on key variables, that the fMRI tasks and manipulations functioned as designed, and that our measures were sensitive to neuropsychological deficits in the reading impaired and the strengths in the non-verbally gifted.

### fMRI data

**Figure 2:**
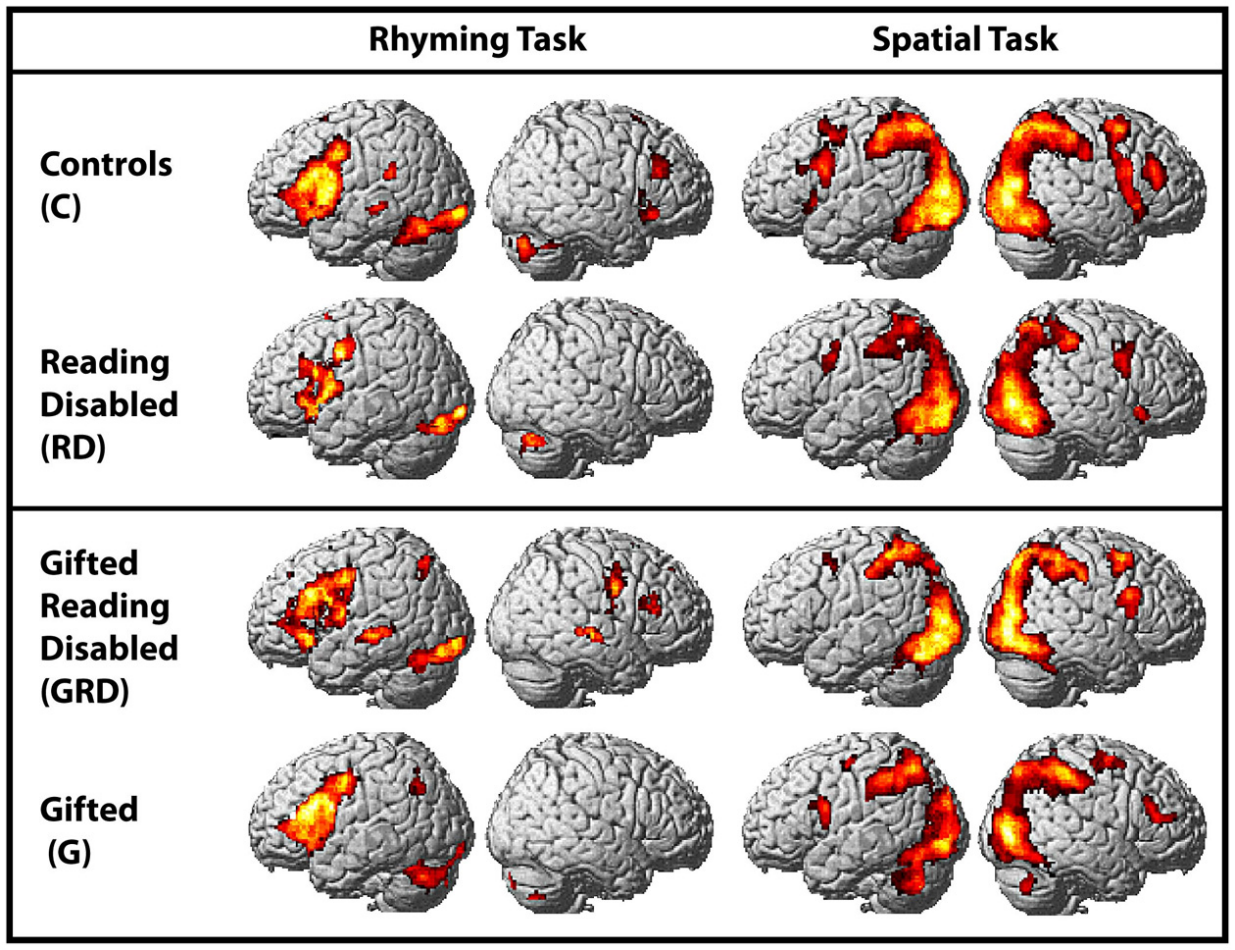
**Activations induced in the whole-brain for within-group comparisons for the word and spatial assessments (also see Table 4).** Columns 1 and 2: Within group activation maps for the word reading task. Significant clusters observed in left inferior frontal and occipitotemporal regions. Columns 3 and 4: Within group activation maps for the spatial task. All maps are presented at a threshold of *p* < 0.001; cluster-size corrected for multiple comparisons.

Within-group whole-brain activation maps are presented in Figure 2, and the parameters of activation peaks are presented in Table 3. While our discussion below focuses on the analyses of regions exhibiting reliable differences between groups, we present Figure 2 and region coordinates for the reader's interest, and to demonstrate that our tasks elicit activity in expected brain areas [i.e., the left inferior frontal and occipitotemporal regions for the reading tasks—Pugh et al. (2001); and in bilateral parietal and middle frontal regions for the spatial assessment tasks—O'Boyle et al., 2005]. We touch upon this further in the discussion.

**Table 3:** **Location of activation peaks—within group comparisons**.

**MNI Co-ordinates**								**MNI Co-ordinates**							
**Task**	**Group**	***x***	***y***	***z***	**Anatomical region**	**BA^1^**	***Z*^2^**	**Group**	***x***	***y***	***z***	**Anatomical region**	**BA^1^**	***Z*^2^**
Rhyming > Line	Controls	−42	−56	−26	L. Cerebellum	^*^	6.33	Reading disabled	−28	−96	−12	L. Inferior occipital gyrus	18	5.23
		−50	−42	22	L. Inferior parietal lobule	13	4.91		−36	28	2	L. Inferior frontal gyrus	47	5.31
		−60	−28	−4	L. Middle temporal gyrus	21	4.42		−2	6	68	L. Superior frontal gyrus	6	5.44
		−4	18	54	L. Superior frontal gyrus	6	6.76							
		−40	10	24	L. Inferior frontal gyrus	9	7.49							
		10	−82	−32	R. Cerebellum	^*^	4.88		22	−66	−24	R. Cerebellum	^*^	4.29
		44	−76	−38	R. Cerebellum	^*^	5.13							
		46	34	34	R. Middle frontal gyrus	9	4.22							
		60	14	6	R. Insula	47	4.23							
	Gifted	−42	−62	−32	L. Cerebellum	^*^	6.12	Gifted RD	−36	−80	−16	L. Inferior occipital gyrus	18	5.70
		−36	−62	36	L. Angular gyrus	39	5.00		−24	−64	52	L. Superior parietal lobule	7	5.30
		−48	−2	46	L. Inferior frontal gyrus	9	7.55		−60	−30	−2	L. Middle temporal gyrus	21	6.00
		−6	16	46	L. Medial frontal gyrus	8	6.12		−36	26	−4	L. Inferior frontal gyrus	9	7.13
		20	−88	−34	R. Cerebellum	^*^	4.96		56	−22	−4	R. Middle temporal gyrus	21	5.21
		28	−66	−48	R. Cerebellum	^*^	5.01		6	−20	60	R. Medial frontal gyrus	6	5.01
									54	−4	34	R. Precentral gyrus	6	5.40
									56	26	24	R. Inferior frontal gyrus	46	4.72
Spatial > Line	Controls	−22	−64	52	L. Precuneus	7	7.84	Reading Disabled	−36	−82	−12	L. Inferior Occipital Gyrus	19	6.72
		−30	22	−4	L. Inferior frontal gyrus	47	5.57		−44	−2	46	L. Middle frontal gyrus	6	4.26
		−50	8	26	L. Middle frontal gyrus	6	6.25							
		18	−74	54	R. Precuneus	7	7.09		38	−80	0	R. Middle occipital gyrus	19	7.73
		28	−2	52	R. Middle frontal gyrus	6	7.41		30	22	−6	R. Inferior frontal gyrus	47	4.61
		6	18	52	R. Medial frontal gyrus	32	6.29		14	20	50	R. Superior frontal gyrus	8	3.99
									48	8	28	R. Inferior frontal gyrus	9	5.01
	Gifted	−38	−48	36	L. Inferior parietal lobule	40	7.65	Gifted RD	−36	−70	−16	L. Middle occipital gyrus	19	6.84
		−24	−16	60	L. Middle frontal gyrus	6	6.55		−22	4	52	L. Middle frontal gyrus	6	5.49
		−46	6	20	L. Inferior frontal gyrus	9	5.51							
		28	−78	10	R. Middle occipital gyrus	19	7.21		42	−80	14	R. Middle occipital gyrus	19	7.07
		28	−66	−40	R. Cerebellum	^*^	5.00		2	−44	−46	R. Cerebellum	^*^	5.03
		28	−16	54	R. Middle frontal gyrus	6	5.18		50	8	26	R. Inferior frontal gyrus	9	4.56
		42	30	20	R. Middle frontal gyrus	46	4.50		34	2	50	R. Middle frontal gyrus	6	5.56

1 *BA: Nearest Brodmann Cytoarchitectonic Area to location of peak activation*.

2 *Z-score representing statistical significance of activity at peak location*.

* *indicates no BA available for this region*.

**Figure 3:**
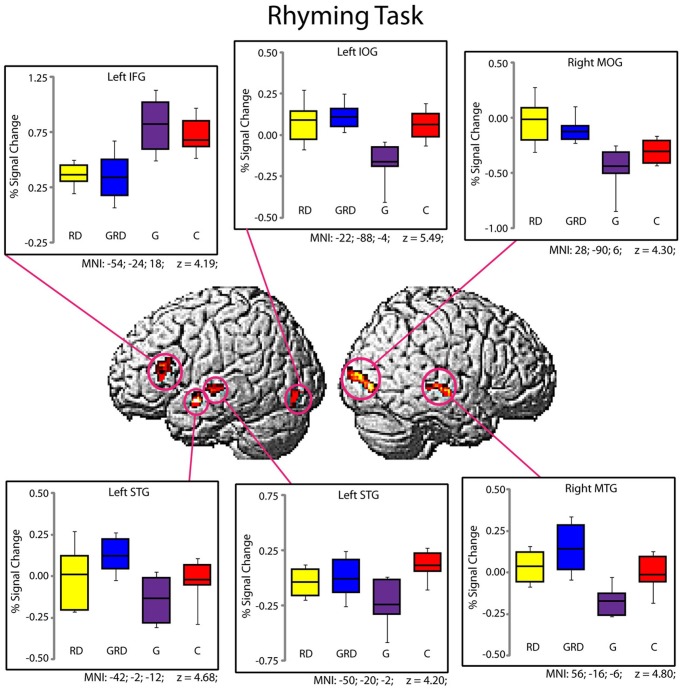
**Activity within the significant clusters from the main effect of Group for the Rhyming tasks (also see Table 4).** Significantly active clusters (*p* < 0.001; cluster-size corrected) were surface rendered onto the SPM MNI template and represent regions that exhibited reliable differences in activation between the four groups. Boxplots represent percent signal change within the specific clusters for the four groups (RD: yellow, GRD: blue, G: purple, C: red). Horizontal lines within the boxplots represent the average percent signal change for W and NW tasks. First and third quartiles are defined by the box edges, and whiskers define the10th and 90th percentiles. MNI co-ordinates of the peak location within the clusters are presented below each corresponding boxplot and in Table 4. Patterns of activation were generally similar for RD and GRD groups.

**Figure 4:**
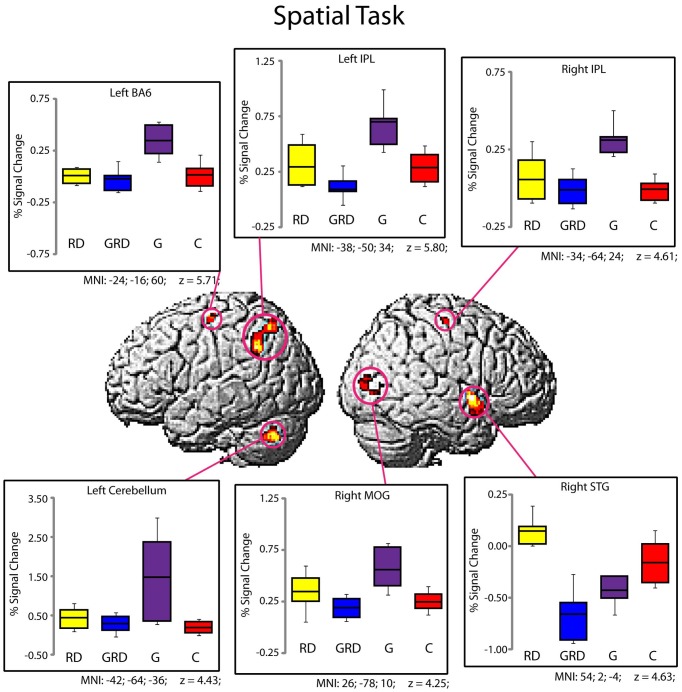
**Activity within the significant clusters from the main effect of Group for the Spatial Visualization tasks (also see Table 4).** Significantly active clusters (*p* < 0.001; cluster-size corrected) were surface rendered onto the SPM MNI template and represent regions that exhibited reliable differences in activation between the four groups. Boxplots represent percent signal change within the specific clusters for the four groups (RD: yellow, GRD: blue, G: purple, C: red). Horizontal lines within the boxplots represent the average percent signal change for R and NR tasks. First and third quartiles are defined by the box edges, and whiskers define the 10th and 90th percentiles. MNI co-ordinates of the peak location within the clusters are presented below each corresponding boxplot and in Table 4. Overall patterns of activation were similar for RD and GRD groups, and significant differences appeared to be driven by greater activation levels for the G group compared to the others.

Significantly active clusters exhibiting a main effect of *Group* from the ANOVA are presented in Figure 3 for the reading assessment, and in Figure 4 for the spatial assessment. In each case, the mean percent signal change within the cluster is plotted for each of the four groups. MNI co-ordinates and anatomical locations of these clusters are presented in Table 4.

**Table 4:** **ANOVA Indicated activation peaks for word and spatial tasks**.

**Hemisphere**	**Region**	**Z-Score**	**Coordinates**	**Brodmann area**
**RHYMING TASK**				
Left	Inf. Frontal Gyrus	4.19	−54 24 18	BA45
	Inf. Occipital Gyrus	5.49	−22 −88 −4	BA17
	Sup. Temporal Gyrus	4.68	−42 −2 −12	BA21
	Sup. Temporal Gyrus	4.20	−50 −20 −2	BA22
Right	Mid. Occipital Gyrus	4.30	28 −90 6	BA18
	Mid. Temporal Gyrus	4.80	56 −16 −6	BA21
**SPATIAL TASK**				
Left	Inf. Parietal Lobule	5.80	−38 −50 34	BA40
	Cerebellum	4.43	−42 −64 −36	^*^
	Precentral Gyrus	5.71	−24 −16 60	BA6
Right	Inf. Parietal Lobule	4.61	34 −64 24	BA40
	Mid. Occipital Gyrus	4.25	26 −78 10	BA18
	Sup. Temporal Gyrus	4.63	54 2 −4	BA22

* *indicates no BA available for this region*.

#### Group differences for rhyming

Five distinct regions (six clusters) exhibited significant differences between the four groups for the rhyming task (Table 4). These included two left hemisphere language regions: the STG and the IFG, as well as the left inferior occipital gyrus (IOG). Two clusters were observed in the left STG, and here, activity did not differ between the GRD and RD groups (*p* > 0.3 for both clusters). However, the GRD group exhibited significantly greater activation than the G group in the more anterior cluster (*p* = 0.013). A similar result was observed in the left IOG: here again, the RD and GRD groups exhibited comparable activation levels (*p* > 0.7), but both were significantly more active than the G group (*p* = 0.002). Notably, in the left IFG, the RD and GRD groups again exhibited similar activation levels (*p* > 0.8), but lower activation relative to the G (*p* = 0.012) and C (*p* = 0.023) groups, providing evidence for relative under-activation for RD individuals often reported in this region. In the right hemisphere, two regions exhibited significant between-group differences: the middle occipital gyrus (MOG) and the middle temporal gyrus. In both cases, activity for the GRD group was similar to the RD group (*p* > 0.2), but was higher than both the G group (*p* < 0.002 in both regions) and the C group (*p* < 0.03 in both regions). This may represent a form of right hemisphere compensation sometimes reported for RD individuals (see Discussion). Overall, for all regions exhibiting group differences for the rhyming tasks, activity for the GRD group was similar to the RD individuals, and not the G individuals.

#### Group differences for spatial visualization

Figure 4 and Table 4 depict six regions where the groups differed significantly for the spatial task: the left Precentral Gyrus/BA6, left cerebellum, bilateral inferior parietal lobule (IPL), right MOG, and the right STG. In five of these six regions (all regions except the right STG), brain activity did not differ between RD and GRD groups (*p* > 0.1). In these regions, the activation levels were comparable between these groups and the control group. Furthermore, activity for these groups in these regions was less than for the G group (*post-hoc* comparisons: G vs. GRD: Left BA6, Left IPL, Right IPL: *p* = 0.001; Right MOG: *p* = 0.002; Left Cerebellum: *p* = 0.026). Thus, group differences appear primarily driven by significantly greater activity for the G group relative to all others, and as with the rhyming assessment, GRD activation levels were similar to the RD group, but primarily differed from the G group for the spatial task. We did observe a different pattern of activity in the right STG. Here, the RD group exhibited the highest activation, which was significantly greater than for the GRD group (*p* < 0.001). Furthermore, in this region, the GRD and G groups exhibited activation levels that did not significantly differ.

## Discussion

The main goal of this study was a preliminary fMRI exploration of if (and how) the GRD brain differs in functionality compared to RD and G brains. As noted in the Introduction, this goal was framed in the context of three hypotheses: 1. *GRD brain activation patterns resemble an admixture of gifted (G) and RD brains*, 2. *GRD brains activate similarly to RD brains (or conversely like G brains)*, or 3. *GRD brains are unique brains and activate in ways that are deviant from G or RDs alone or in combination*. Our approach to the analysis was to focus on the regions that demonstrated significant differences in the omnibus ANOVA on the four subject groups for the reading (rhyming) and spatial tasks, and then to perform subsequent *post-hoc* comparisons to determine whether activity for the GRD group in these regions was comparable to either the RD group or the G group.

First, it is noteworthy that in many ways the performance of the C group replicates prior research on reading-related and spatial tasks (see Figures 2–4, Tables 3, 4), with areas of significant activation for word reading in the left inferior frontal, superior temporal and inferior occipital regions (Pugh et al., 2001; Price et al., 2003), and activation for spatial processing in bilateral areas including the left and right IPL, right MOG and STG, and the cerebellum (Cohen et al., 1996; O'Boyle et al., 2005; Gogos et al., 2010). For the rhyming task, the RD group often exhibited underactivation relative to the C group (Shaywitz et al., 2002), and a previous report comparing only the C vs. RD groups (Olulade et al., 2012) also demonstrated lower activity during word reading as well as spatial processing in the RD sample relative to Cs (i.e., left temporal, frontal and parietal regions during word reading and frontal and parietal areas during spatial visualization). In the present study, we demonstrate that for this same spatial task, the G group often exhibited greater activation than C and/or the other 3 groups, and across multiple regions as expected (O'Boyle et al., 2005).

### Which hypothesis best fits the data?

First, with some qualification, the activation patterns in the GRD group do not appear unique (hypothesis 3). In fact, visual inspection of the patterns of activation above or below baseline (Figures 3, 4) suggests that the GRD brains function most like RD brains. *Post-hoc* pairwise comparisons generally support this claim as well.

Considering the direction of activation relative to baseline, and mean activation and standard errors per region, the GRD pattern for word reading is quite similar to the RD pattern, and at the same time very different than the patterns exhibited by the G and C groups. The clearest deviation from the RD pattern was that the GRDs showed relatively greater activation in the left STG than the other 3 groups. A similar trend was found for the spatial task data: the direction and mean activation levels of the GRD group best fit those of the RD group, although the fit may not be as defined as it was in the case of word reading. One clear deviation from the RD-GRD pattern association was in the right STG, where activity in the RD group was elevated relative to both the GRD and G subjects.

The similarity of RD-GRD fMRI activation patterns is mirrored in the similarity of RD-GRD psychometric test data shown in Table 1. With the exception of our group formation variable, PIQ, RD and GRD subjects test scores were quite similar in reference to Cs. Thus, considering the fMRI and behavioral data together, our conclusion is that the functionality of the GRD brain in adults of our sample is much like that of the RD brain (hypothesis 2). Indeed, there was no evidence that the functional neurology of GRD subjects represented a simple admixture of RD and G activation (hypothesis 1).

### What does similar RD-GRD activation tells us about etiology?

This is the first imaging report examining GRD individuals, and additional research is required. Further determination of which of the three hypothetical options outlined in the Introduction is valid will depend on carefully conducted genetic, epidemiologic and neuroscientific research in the future. However, at this time, we believe that hypothesis 2 fits our data well, and that hypotheses 1 and 3 can likely be rejected given that the GRD subjects were very similar to the RD subjects on psychometrics and for neural responses.

It is important to recognize that our conclusions may apply only to our group of GRD subjects, and it does not directly address whether or not a shared etiology for giftedness and RD exists in the broader RD population as some have suggested. Different research designs are needed to address that issue. However, because the shared etiology issue is an important and historical concern, it is noteworthy that our data do not rule out this mechanism. For example, if non-verbal giftedness shares an etiology with RD in our GRD group, the two conditions have interacted throughout the lifespan, with one possible consequence being that a high PIQ, or a high potential PIQ, has helped the individual with RD compensate for linguistic problems and academic performance from childhood to adulthood. This compensatory reliance on right and left hemisphere functions that originally had high potential (for example high visuo-spatial abilities) may limit or modify their expression in ways we observed in the psychometric and functional patterns. Indeed, our data are consistent with much of the literature showing that adult RD subjects do not necessarily perform significantly above average on tasks of spatial analysis or processing of various sorts, although they may *approach* such tasks differently than non-RD subjects (Winner et al., 2001; Olulade et al., 2012). That is, their external behavior may not appear exceptional, but the internal neural computation used to perform the tasks may be different. Speculatively, if such activation differences exist, they could be a sort of “residual” footprint that our RD and GRD adults do naturally approach spatial problems in unique ways, but their expression of this difference has changed over development as the deficit disability has interacted with the higher ability.

There is in fact evidence in our fMRI data that subjects may have compensated for their reading problems: Areas of right hemisphere (MTG, MOG) maintain an importance in word processing in these RD and GRD college students that is unlike that in degree or direction compared to normally reading students. An over reliance on these right hemisphere areas for reading has been shown in RD individuals, and a shift from the right to the left hemisphere reading areas is indicated in response to remediation (Simos et al., 2002). In our samples, the right MOG was the only region that was significantly different among the 4 groups for both the word and spatial MRI tasks. The G group exhibited strong deactivation relative to baseline during reading and increased activation during spatial processing, whereas the GRD (and RD) groups did not deactivate this area to the same degree during reading, and both groups were relatively under-activated relative to the G group in this region during the spatial task. Other research has shown that the right MOG has a preferential activation for spatial information and a deactivation for auditory information regardless of experience (Renier et al., 2010). This pattern is seen in our data. However, the GRD group does not activate or deactivate the right MOG to the same degree as seen in their G counterparts. Furthermore, all 4 groups activated the left IFG during reading, although activation was strongest for the C and G subjects. Consistent with prior research, a strong reliance on the IFG, along with an over reliance on other non-typical reading areas by RD college students probably indicates the effects of experience and compensation (Hoeft et al., 2011).

Another region that was active for both the word and spatial MRI tasks was the STG, although it was left sided for the word task and right sided for the spatial task. Interestingly the STG is the region that the GRDs and the RDs most differed on: during the word task, the GRD activation in the left STG was unique from the other 3 groups; during the spatial task, the GRD pattern was similar to the G for the right STG. While the STG results may suggest that the GRDs represent a unique etiologic subtype of RD (*hypothesis 3*), it is also possible that the left and right STG hemispheric homologues are interacting at different stages during the processing of the phonological aspects of the word task (Bitan et al., 2010), and the greater activation of the GRDs in the left STG (around Wernicke's area) is an effect of an experientially practiced increase in connectivity where original strengths in the right STG help compensate for reading deficits via the left STG. Nevertheless, deactivation of the right STG appears important in the processing of spatial information in our C, GRD, and G subjects. In addition to auditory-linguistic processing, the right STG and its cortical and sub-cortical networks have been implicated in a variety of non-verbally-oriented functions (Karnath, 2001).

### Concluding remarks and caveats

The present study suggests that non-verbally gifted RD adults resemble non-gifted RD adults in their performance on paper-and-pencil reading, math and spatial tests, and in their patterns of functional activation during reading and spatial processing. In our opinion, a shared etiology of RD and giftedness can yield a lifespan interaction with reading compensation effects modifying how an adult brain processes text and spatial stimuli. In fact, the very nature of learning how to read English at an early age may “interfere” with how well the brain deals with visual-spatial stimuli relative to readers of more visual/orthographic text formats (McBride-Chang et al., 2011).

It is important to bear in mind that our 4 subject groups have true and statistically reliable differences on the reading and PIQ selection criteria, but they may not represent their population counterparts accurately. Additional work is needed, particularly with younger ages, using different tasks, group definitions and methods. The interpretation of these results is also limited by sample size, although our sizes are not deviant from many other MRI studies of clinical and uncommon populations, and significant effects were observed. Still, we are at risk of making beta errors, or not detecting a difference in our sample that may exist in the population. Thus, we hope that this preliminary data will inspire other projects of larger design to address this possibility. Finally, we did not control for the rate and possibility of Type I errors in our *t*-tests of the psychometric data (yet the fMRI results presented here survived strong statistical thresholds including corrections for multiple comparisons). Given our small sample, corrections for multiple comparisons seemed overly restrictive, as this was a first-time exploration into this area of research. Again, generalizing from our results should be done with caution and replication studies are needed.

Beyond the obvious value to our understanding of lifespan neurodevelopment, work in this area has important implications for diagnosis and treatment. For instance, there currently exists great interest in examining the potential for spatial skill development (rather than ultimate aptitude) in young children with RD, whether this potential differs from that in controls or changes with age, and, perhaps, if such potential can be fostered alongside common reading remediation practices. Studies such as the current one represent a crucial first step in providing answers to these questions.

### Conflict of interest statement

The authors declare that the research was conducted in the absence of any commercial or financial relationships that could be construed as a potential conflict of interest.

## Footnotes

^1^While we refer to our G and GRD groups as “gifted,” we recognize that in practice these individuals may not reach the threshold for a gifted classification based on a standardized test. Our average nonverbal IQ in our samples is technically in the high/superior range (>120) and this may not meet the cut-off criteria for some gifted programs or reach the standard range for giftedness on common IQ tests (>130). However, all GRD and G adults have high/superior IQs in the nonverbal domain that stand out from their normal performance in the verbal domain or their deficits in reading. Clearly these subjects had strengths in the nonverbal area and many subjects reported talents or hobbies in the arts, math and other spatially-oriented applications. Furthermore, many of the GRD subjects reported common issues seen in twice exceptional students with nonverbal strengths, such as complicated diagnoses in elementary school, splinter skills and a preference for nonreading/nonverbal activities. The PIQ > VIQ split in the GRD group is also a frequently observed profile in the twice exceptional. For these reasons we feel comfortable using our G and GRD comparison groups as adequate representatives of gifted and twice exceptional samples, albeit they are not in the extreme gifted category and future research on such groups would further advance the field.

^2^The notable exceptions to this statement were that NR stimuli elicited less activation than R stimuli for the G and GRD groups, and W and NW stimuli while generally under activated in the RD group, elicited activity in different frontal lobe regions. That said, the group analyses combining within task stimulus types led to more coherent results that did not differ significantly from analyses performed separately on the within task stimulus types. Data on W and NW, and R and NR activation patterns are available from the authors.

## References

[B1] Bitan T. Lifshitz A. Breznitz Z. Booth J. R. (2010). Bidirectional connectivity between hemispheres occurs at multiple levels in language processing but depends on sex. J. Neurosci. 30, 11576–11585 10.1523/JNEUROSCI.1245-10.2010 20810879 PMC2943218

[B2] Bryden M. P. MacManus I. C. Bulman-Fleming M. B. (1994). Evaluating the empirical support for the Geschwind-Behan-Galaburda model of cerebral lateralization. Brain Cogn. 26, 103–167 10.1006/brcg.1994.1045 7531983

[B3] Cohen M. S. Kosslyn S. M. Breiter H. C. DiGirolamo G. J. (1996). Changes in cortical activity during mental rotation: a mapping study using functional MRI. Brain 119, 89–100 10.1093/brain/119.1.89 8624697

[B4] Craggs J. Sanchez J. Kibby M. Gilger J. Hynd G. (2006). Brain morphology and neuropsychological profiles in a family displaying dyslexia and superior nonverbal intelligence. Cortex 42, 1107–1118 10.1016/S0010-9452(08)70222-3 17209416

[B5] Eide B. Eide F. (2006). The Mislabeled Child: How Understanding Your Child's Unique Learning Style Can Open the Door to Success. New York, NY: Hyperion

[B6] Foley Nicpon M. Allmon A. Sieck B. Stinson R. (2011). Empirical investigation of twice-exceptionality: where have we been and where are we going? Gifted Child Q. 55, 3–17 10.1177/0016986210382575

[B7] Friston K. J. Glaser D. E. Henson R. N. A. Kiebel S. Philips C. Ashburner J. (2002). Classical and bayesian inference in neuroimaging: applications. Neuroimage 16, 484–412 10.1006/nimg.2002.1091 12030833

[B8] Galaburda A. M. (1992). Neurology of developmental dyslexia. Curr. Opin. Neurol. Neurosurg. 5, 71–76 1623242

[B9] Geschwind N. Behan P. (1982). Left handedness: association with immune disease, left handedness, and developmental learning disorder. Proc. Natl. Acad. Sci. U.S.A. 79, 5097–5100 10.1073/pnas.79.16.5097 6956919 PMC346835

[B10] Geschwind N. Galaburda A. M. (1987). Cerebral Lateralization: Biological Mechanisms, Associations and Pathology. Cambridge, MA: MIT Press

[B11] Gilger J. Hynd G. (2008). Neurodevelopmental variation as a basis for thinking about the twice exceptional. Roeper Rev. 30, 214–228 10.1080/02783190802363893

[B12] Gogos A. Gavrilescu M. Davison S. Searle K. Adams J. Rossell S. L. (2010). Greater superior than inferior parietal lobe activation with increasing rotation angle during mental rotation: an fMRI study. Neuropsychologia 48, 529–535 10.1016/j.neuropsychologia.2009.10.013 19850055

[B13] Hoeft F. McCandiss B. D. Black J. Gantman A. Zakerani N. Hulme C. (2011). Neural systems predicting long term outcome in dyslexia. Proc. Natl. Acad. Sci. U.S.A. 108, 361–366 10.1073/pnas.1008950108 21173250 PMC3017159

[B14] Karnath H.-O. (2001). New insights into the functions of the superior temporal cortex. Nat. Rev. Neurosci. 2, 568–576 10.1038/35086057 11484000

[B15] Lerner J. (1989). Educational intervention in learning disabilities. J. Am. Acad. Child Adolesc. Psychiatry 28, 326–331 10.1097/00004583-198905000-00004 2661524

[B16] Logan J. (2009). Dyslexic entrepreneurs: the incidence; their coping strategies and their business skills. Dyslexia 15, 328–346 10.1002/dys.388 19378286

[B17] McBride-Chang C. Zhou Y. Cho J.-R. Aram D. Levin I. Tolchinsky L. (2011). Visual spatial skill: a consequence of learning to read? J. Exp. Child Psychol. 109, 256–262 10.1016/j.jecp.2010.12.003 21237468

[B18] McClain M. C. Pfeiffer S. (2012). Identification of gifted students in the United States today: a look at state definitions, policies, and practices. J. Appl. Sch. Psychol. 28, 59–88 10.1080/15377903.2012.643757

[B19] McManus I. C. Bryden M. P. (1991). Gechwind's theory of cerebral lateralization: developing a formal, causal model. Psychol. Bull. 110, 237–253 10.1037/0033-2909.110.2.237 1946868

[B20] Newman T. Sternberg R. (2004). Students with Both Gifts and Learning Disabilities: Identification, Assessment, and Outcomes. New York, NY: Springer 10.1007/978-1-4419-9116-4

[B21] O'Boyle M. W. Cunnington R. Silk T. J. Vaughan D. Jackson G. Syngeniotis A. (2005). Mathematically gifted male adolescents activate a unique brain network during mental rotation. Cogn. Brain Res. 25, 583–587 10.1016/j.cogbrainres.2005.08.004 16150579

[B22] Olulade O. A. Gilger J. W. Talavage T. M. Hynd G. W. McAteer C. I. (2012). Beyond phonological processing deficits in adult dyslexics: atypical fMRI activation patterns for spatial problem solving. Dev. Neuropsychol. 37, 617–635 10.1080/87565641.2012.702826 23066939

[B23] Pugh K. R. Mencl W. E. Jenner A. R. Katz L. Frost S. J. Lee J. (2001). Neurobiological studies of reading and reading disability. J. Commun. Disord. 34, 479–492 10.1016/S0021-9924(01)00060-0 11725860

[B24] Pugh K. R. Mencl W. E. Shaywitz B. A. Shaywitz S. E. Fulbright R. K. Constable R. T. (2000). The angular gyrus in developmental dyslexia: task-specific differences in functional connectivity within posterior cortex. Psychol. Sci. 11, 51–56 10.1111/1467-9280.00214 11228843

[B25] Price C. J. Gorno-Tempini M.-L. Graham K. S. Biggio N. Mechelli A. Patterson K. (2003). Normal and pathological reading: converging evidence from lesion and imaging studies. Neuroimage 20, s30–s41 10.1016/j.neuroimage.2003.09.012 14597294

[B26] Renier L. A. Anurova I. De Volder A. G. Carlson S. VanMeter J. Rauschecker J. P. (2010). Preserved functional specialization for spatial processing in the middle occipital gyrus of the early blind. Neuron 68, 138–148 10.1016/j.neuron.2010.09.021 20920797 PMC2951740

[B27] Ruban L. M. Reis S. M. (2005). Identification and assessment of gifted students with learning disabilities. Theory Into Practi. 44, 115–124 10.1207/s15430421tip4402_6

[B28] Schneps M. H. Rose L. T. Fisher K. W. (2007). Visual learning and the brain: implications for dyslexia. Mind Brain Educ. 1, 128–139 10.1111/j.1751-228X.2007.00013.x

[B29a] Shaywitz S. E. Shaywitz B. A. Pugh K. R. Fulbright R. K. Constable R. T. Mencl W. E. (1998). Functional disruption in the organization of the brain for reading in dyslexia. Proc. Natl. Acad. Sci. 95, 2636–2641 10.1073/pnas.95.5.2636 9482939 PMC19444

[B29] Shaywitz B. A. Shaywitz S. E. Pugh K. R. Mencl W. E. Fulbright R. K. Skudlarski P. (2002). Disruption of posterior brain systems for reading in children with developmental dyslexia. Biol. Psychiatry 52, 101–110 10.1016/S0006-3223(02)01365-3 12114001

[B30] Shepard R. N. Metzler J. (1971). Mental rotation of three-dimensional objects. Science 171, 701–703 10.1126/science.171.3972.701 5540314

[B31] Simos P. G. Fletcher J. M. Bergman E. Breier J. I. Foorman B. R. Castillo E. M. (2002). Dyslexia-specific brain activation profile becomes normal following successful remedial training. Neurology 58, 1203–1213 10.1212/WNL.58.8.1203 11971088

[B32] Wechsler D. (1999). Wechsler Abbreviated Scale of Intelligence (WASI). San Antonio, TX: The Psychological Corporation

[B33] West T. G. (1999). The abilities of those with reading disabilities: focusing on the talents of people with dyslexia, in Reading and Attention Disorders: Neurobiological Correlates, ed Duane D. D. (Baltimore, MA: York Press), 213–241

[B34] Wilkinson G. S. Robertson G. J. (2006). Wide Range Achievement Test-Fourth Edition. Lutz, FL: Psychological Assessment Resources

[B35] Winner E. von Karolyi C. Malinsky D. French L. Seliger C. Ross E. (2001). Dyslexia and visual-spatial talents: compensation vs. deficit model. Brain Lang. 76, 81–110 10.1006/brln.2000.2392 11254251

[B36] Wolf M. Denckla M. B. (2005). Rapid Automatized Naming and Rapid Automatized Stimulus Tests. Austin, TX: Pro-Ed

[B37] Wolfe L. E. Schreiber H. E. Wasserstein J. (2008). Adult Learning Disorders: Contemporary Issues. New York, NY: Psychology Press, Taylor and Francis Group

[B38] Woodcock R. W. McGrew K. S. Mather N. (2001). Woodcock-Johnson III Tests of Achievement. Itasca, IL: Riverside Publishing

